# Cardiomyocyte proliferation: Advances and insights in macrophage-targeted therapy for myocardial injury

**DOI:** 10.1016/j.gendis.2024.101332

**Published:** 2024-05-19

**Authors:** Tao Wang, Xueyao Wang, Weibin Ren, Zeyu Sun, Yanhui Zhang, Nanping Wu, Hongyan Diao

**Affiliations:** a Jinan Microecological Biomedicine Shandong Laboratory, Jinan, Shandong 250117, China; b State Key Laboratory for Diagnosis and Treatment of Infectious Diseases, National Clinical Research Center for Infectious Diseases, National Medical Center for Infectious Diseases, Collaborative Innovation Center for Diagnosis and Treatment of Infectious Diseases, The First Affiliated Hospital, Zhejiang University School of Medicine, Hangzhou, Zhejiang 310003, China

**Keywords:** Cardiac regeneration, Cardiomyocyte proliferation, Heterogeneous macrophages, Macrophages, Myocardial infarction

## Abstract

In the mammalian heart, cardiomyocytes undergo a transient window of proliferation that leads to regenerative impairment, limiting cardiomyocyte proliferation and myocardial repair capacity. Cardiac developmental patterns exacerbate the progression of heart disease characterized by myocardial cell loss, ultimately leading to cardiac dysfunction and heart failure. Myocardial infarction causes the death of partial cardiomyocytes, which triggers an immune response to remove debris and restore tissue integrity. Interestingly, when transient myocardial injury triggers irreversible loss of cardiomyocytes, the subsequent macrophages responsible for proliferation and regeneration have a unique immune phenotype that promotes the formation of pre-existing new cardiomyocytes. During mammalian regeneration, mononuclear-derived macrophages and self-renewing resident cardiac macrophages provide multiple cytokines and molecular signals that create a regenerative environment and cellular plasticity capacity in postnatal cardiomyocytes, a pivotal strategy for achieving myocardial repair. Consistent with other human tissues, cardiac macrophages originating from the embryonic endothelium produce a hierarchy of contributions to monocyte recruitment and fate specification. In this review, we discuss the novel functions of macrophages in triggering cardiac regeneration and repair after myocardial infarction and provide recent advances and prospective insights into the phenotypic transformation and heterogeneous features involving cardiac macrophages. In conclusion, macrophages contribute critically to regeneration, repair, and remodeling, and are challenging targets for cardiovascular therapeutic interventions.

## Introduction

Cardiomyocytes possess a strong regenerative capacity during embryonic life; however, cardiomyocytes lose their regenerative capacity after birth after a narrow proliferation window, resulting in impaired proliferation.^1^^,^^2^ The inability to replenish functional cardiomyocytes due to impaired regeneration of adult cardiomyocytes directly contributes to the poor prognosis after myocardial injury. Thus, myocardial infarction (MI) as a cardiac disease characterized by myocardial cell loss, exacerbates the process of cardiac dysfunction and heart failure. Depending on the abundance of different cell types in the heart, cardiomyocytes and non-cardiomyocyte populations are distributed throughout the heart in certain proportions. Although cardiomyocytes occupy most of the space of the heart, the heart accounts for only 30%–40% of the total cell population.^3^ Genetic tools and immunohistochemical analyses showed that endothelial cells accounted for more than 60% of non-cardiomyocytes, haematopoietic cells accounted for 5%–10%, and fibroblasts accounted for nearly 20%.^4^ Accompanying the developmental characteristics of cardiomyocytes, the immune system provides a variety of cytokines and molecular signals to regulate cardiomyocyte repair and regenerative capacity after birth. Macrophages are the most abundant and ubiquitous immune cells, and self-polarization confers enormous phenotypic and functional plasticity to macrophages, enabling them to act as pro-inflammatory, homeostatic, and anti-inflammatory agents.^5^ Previous studies have demonstrated that MI triggers a strong inflammatory and immune response to remove damaged matrix debris and activates a series of myocardial fibrotic scar repair processes.^6^ During the onset of myocardial injury, multiple immune cells such as neutrophils, monocyte subsets, macrophages, natural killer cells, regulatory T cells, and B cells initiate myocardial inflammatory and repair regulatory networks.^6^^,^^7^ However, there is heterogeneity in the infiltration and differentiation of immune cells at different stages of myocardial injury, and the role of multiple endogenous signaling molecules in the regulation of myocardial inflammation and repair is still being explored.

Macrophages are required for neonatal heart regeneration and efficient neovascularization in 1-day postnatal (P1) mice.^8^ Recent studies have confirmed that embryonic-derived and monocyte-derived macrophages are recruited differently to specific sites after cardiac injury to initiate unique inflammatory and injury repair responses.^9^ Macrophage populations of different origins mediate cardiac inflammatory (M1) and reparative (M2) macrophage phenotypic transitions. Neonatal cardiac-resident macrophages are essential for myocardial regeneration and repair after MI. Phenotypic and distribution dynamics of non-cardiomyocytes show that *CX*_*3*_*CR1*^+^ *CCR2*^*−*^ *Ly6C*^*−*^ *MHCII*^*−*^ (MP1) subclusters directly initiate cardiomyocyte proliferation and myocardial repair via the Jagged-1/Notch1 axis during embryonic day 18 (E18) and P7.^10^ The MP1 subpopulation is primarily responsible for cardiac development and regeneration, including activation of cell mitosis, cell differentiation, and angiogenesis. Interestingly, embryonic-derived macrophages would trigger myocardial proliferation in neonatal hearts, leading to reduced myocardial fibrosis and increased coronary angiogenesis.^11^ However, monocyte-derived macrophages in the injured adult heart induce a strong inflammatory phenotype, leading to diminished angiogenesis and tissue damage, limiting the ability of the adult heart to regenerate and repair. Meanwhile, M1 or M2-polarized macrophages play a crucial role in the proliferation, differentiation, and maturation of induced pluripotent stem cells (iPSCs), providing a theoretical basis for the study of the molecular mechanisms of myocardial proliferation and regeneration.^12^ Notably, macrophages have more than simple immune cell characteristics. They also play a crucial role in tissue development, remodeling, angiogenesis, and metabolism. Macrophages act as a cellular transducer, converting energy from one form to another, sensing microenvironmental stimuli, and initiating cardiomyocyte proliferation and repair potential.^13^ Thus, when transient MI triggers irreversible myocardial cell loss, the subsequent immune inflammatory response weakly compensates for functional myocytes in the form of myocyte proliferation and is a key strategy to achieve myocardial repair. In conclusion, understanding the immune regulation of the heart can help unravel the mechanisms of impaired cardiomyocyte proliferation in the developing heart.

## Origin and functions of cardiac macrophages

**Figure 1:**
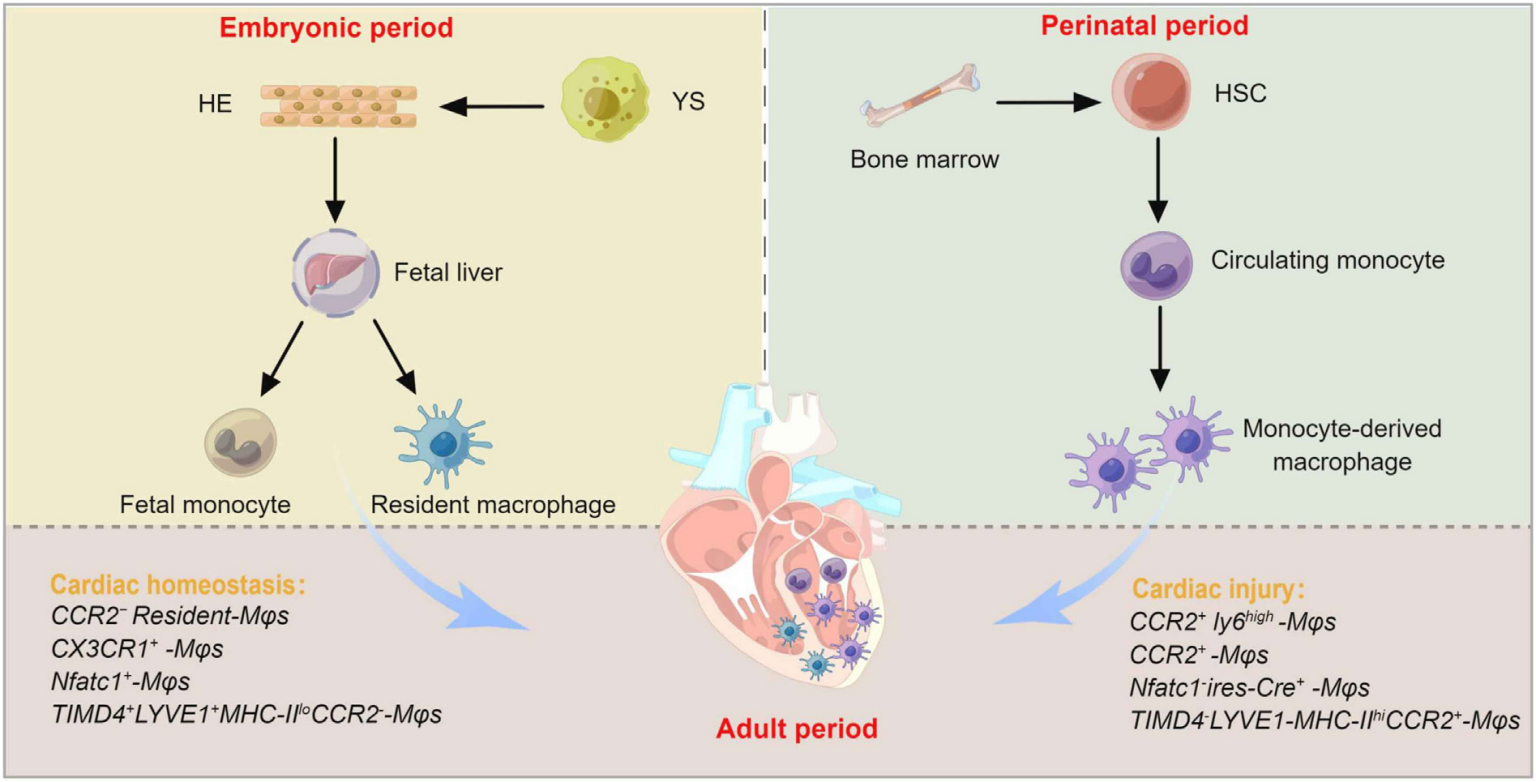
Developmental origin and subtype function of cardiac macrophages. Embryonic development (E7.5-E12.5) revolves around the hematopoietic endothelium (HE) of the yolk sac (YS), which undergoes three waves of hematopoiesis. A short period of deterministic hematopoietic cells migrates to the fetal liver after generating YS macrophages or multiple lineages, which subsequently develop into resident macrophages and eventually migrate to the embryonic heart. During the perinatal period, traditional bone marrow hematopoietic stem cells (HSCs) will become the primary site of hematopoiesis, differentiating into circulating monocytes and macrophages in the peripheral blood to produce a full immune lineage. In adult mice, resident cardiac macrophages are depleted after myocardial infarction and mononuclear-derived macrophages are recruited and enriched in large numbers and subsequently partially restored to the level of the uninjured state.

Understanding the origin and fate of cardiac macrophages plays an important role in cardiac tissue homeostasis and cardiovascular diseases. In the human circulatory system, cardiac-resident macrophages are involved in cardiac growth and development, myocardial healing, coronary blood vessels, and normal cardiac conduction.^14^^,^^15^ In addition, cardiac macrophages conduct electrical conduction in the distal atrioventricular node by expressing connexin-43 and coupling to cardiomyocytes in the contracted state.^16^ Therefore, a deficiency of cardiac macrophages would trigger atrioventricular conduction disorders. Emerging genetic lineage tracing systems are key to constructing fate maps for various cell populations.^17^ Fate mapping localization analysis will attempt to re-evaluate the potential contribution of cardiac hematopoietic cells in cardiac development. In this ongoing study, genetic lineage tracing and immunological developments have further defined the origin of cardiac macrophages (Fig. 1).

## Macrophages in the hematopoietic system

The hematopoietic system is one of the first complex tissues to develop in the mammalian concept.^18^ The origin of the adult mammalian blood system remains a topic of lively discussion and intense research. At mouse E10.5, hematopoietic stem cells begin to be generated in the dorsal aorta and are primarily responsible for blood production in adult mice.^19^^,^^20^ Transformation of endothelial cells to hematopoietic cells occurs during primitive and definitive hematopoiesis in mouse embryos by endothelial lineage tracking techniques. Embryonic development revolves around the hematopoietic endothelium of the yolk sac (YS), which undergoes three waves of hematopoiesis. The first primitive wave begins with the aggregation of extraembryonic mesodermal cells on the YS at E7.5 into blood islands, which are the initial hematopoietic centers. Subsequently, the blood islands give rise to early primitive erythroid-like progenitors, which signal the formation of macrophages and microglia. At this stage of development, macrophages are first observed and expand in the extra-embryonic YS, being the only “leukocytes” produced, indicating a conserved origin of the YS-derived macrophage lineage.^21^ During E8.0 and E8.25, transient definitive waves begin to localize to the hematopoietic endothelium of the YS. Immediately afterward, at E8.5∼E9.5, transient definitive hematopoiesis takes place in the fetal liver after generating YS macrophages or multiple lineages. In conclusion, both YS tissue-resident macrophages and fetal monocytes arise from erythroid-like progenitors generated in the YS.^22^ At E11.5∼E12.5, the third wave of decisive hematopoiesis occurs in the para-aortic pleura and the aorta-gonad-midkidney, where hematopoietic stem and progenitor cells colonize the fetal liver and subsequently develop resident macrophages, which eventually migrate to the embryonic heart. In the perinatal period, traditional bone marrow hematopoietic stem cells will become the primary site of hematopoiesis, generating complete immune lineages.^23^ Hematopoietic stem cells in the bone marrow undergo differentiation to produce circulating monocytes and macrophages in the peripheral blood.^24^ Thus, hematopoietic stem and progenitor cells and all blood cell lineages originate specifically from the hematopoietic endothelium. During mammalian embryonic development, hematopoietic stem cells at the top of the hematopoietic system hierarchy can differentiate into all types of blood and immune cells, and understanding the origin of macrophages is important for cardiac development and inflammatory responses.

## Cardiac-derived macrophage subsets

Macrophages are specialized innate immune cells with significant transcriptional differences between different subpopulations of tissues.^25^, ^26^, ^27^ In recent years, studies have uncovered that the heterogeneity of macrophages within and outside of organs contributes to the understanding of biological differences.^28^ Two macrophage subsets of different origins are present in the myocardium, including self-renewing resident macrophages and macrophages derived from monocytes.^29^, ^30^, ^31^ Studies have demonstrated that macrophages from different tissues and organs originate from at least three different lineages.^32^ YS progenitors and fetal monocytes seed tissues during development with bone marrow-derived monocytes contributing after birth, resulting in organ-specific compositions. Genetic fate mapping shows that YS and fetal monocyte progenitor cells give rise to the majority of cardiac macrophages, and the heart is one of the few organs in which large numbers of YS macrophages are still present in adulthood.^33^ To investigate the role of cardiac macrophages in cardiac repair, researchers found that in diphtheria toxin-treated *CD11b*^*DTR*^ mice, genetic deletion of *CD11b*-positive macrophages resulted in impaired neonatal cardiac regeneration after apical resection injury.^34^ Interestingly, transplantation of cardiac macrophages from neonatal mice into adult mice with myocardial injury effectively promoted cardiomyocyte proliferation and myocardial repair.

Unlike neonatal cardiac macrophages, monocyte-derived macrophages predominate in the monocyte-macrophage population after adult myocardial injury.^11^^,^^35^ Filipa et al established that monocyte-derived macrophages influence the key processes of cardiac regeneration. When adult spleen-derived macrophages were transplanted into neonates, impaired regenerative capacity and increased scar formation after MI revealed an anti-regenerative effect of monocytes and their source macrophages. To investigate the individual transformation and diversity of monocyte-derived macrophages during cardiac injury, it is critical to understand the mechanisms regulating monocyte recruitment and fate specification. Through several mouse models of cardiomyocyte death, including permanent MI, reperfusion MI, and diphtheria toxin cardiomyocyte ablation, it was revealed that tissue-resident macrophages were predominantly replaced by infiltrating monocytes and monocyte-derived macrophages.^35^ In cardiac development, Molawi et al identified embryonic-derived cardiac macrophages that showed a decline in self-renewal with age and were replaced by CC chemokine receptor 2 (*CCR2*^*+*^) expressing-macrophages differentiated from peripheral monocytes, even in the absence of inflammation.^36^ In a comparison of heterogeneous macrophage fate norms after myocardial injury, adult mice *CCR2*^+^ macrophages infiltrate infarct sites for prolonged periods and consistently and persistently express high levels of inflammatory cytokines. In contrast, monocyte-derived *CCR2*^+^ macrophages were not recruited after cardiac injury in neonatal mice, and aggregated macrophages expressed low levels of inflammatory factors. Genetic fate mapping shows that cardiac macrophages derived from CX3C chemokine receptor1 (*CX3CR1*) embryonic progenitors persist into adulthood, but the initial high contribution to resident cardiac macrophages declines gradually after birth.^32^^,^^36^, ^37^, ^38^ *CX3CR1*-based system-induced macrophage depletion leads to cardiac dysfunction and poor remodeling, revealing a non-redundant cardioprotective role for cardiac macrophages. In conclusion, cardiac resident macrophages possess potent myocardial proliferation and repair potential and are a promising new tool for targeted therapy for cardiac regeneration. However, further studies are needed to identify more precise targets of intervention and immune rejection for neonatal cardiac macrophage transplantation.

## Lineage tracing of tissue-resident macrophages

Previous studies have shown that approximately 58% of macrophages in endocardial pads were found to be derived from the nuclear factor of activated T-cells 1 (*Nfatc1*)-Cre-labeled endocardium using a genetic lineage tracking system.^39^ However, genetic fate mapping techniques require cell-specific expression of the gene promoter that drives Cre recombinase.^17^^,^^40^^,^^41^ This has led to a degree of confounding of marker genes and the need to re-evaluate the contribution of *Nfatc1*-derived cells to embryonic hematopoiesis. Recent studies have shown that three different *Nfatc*1 knock-in tools, including *Nfatc1*-ires-Cre, *Nfatc1*-2A-Dre, and *Nfatc1*-2A-CreER were used to reassess the endocardial contribution of *Nfatc1*-derived cells to embryonic hematopoiesis.^42^ Data from *Nfatc1*-2adre-mediated tracing suggest that *Nfatc1* is not a specific genetic marker for targeting endocardium and that its tagged endocardium has no *in vitro* hematopoietic potential. Notably, in *Nfatc1*-2A-CreER;R26-tdTomato reporter mice, a subpopulation of cardiac macrophages, circulating macrophages, and monocytes express the Nfatc1 gene. Three possible sources of Nfatc1-labeled cardiac macrophages include the hematopoietic output of *Nfatc1*-labeled hematopoietic endothelium from the YS and the dorsal aorta; *Nfatc1*-ires-Cre directly labeled *Nfatc1*^+^ cardiac macrophages; and circulating *Nfatc1*^+^macrophages and *Nfatc1*-ires-Cre-labeled monocytes. Thus, developing cardiac macrophages are mainly derived from the YS-phase primitive and transient deterministic hematopoietic process endothelial-to-hematopoietic transition. These findings refute the concept of endocardial hematopoiesis and suggest that the developing endocardium rarely produces hematopoietic cells, including cardiac macrophages.

Recent research has found that adult valve fibroblasts may originate from hematopoietic stem cells, and it is the first time to find immune cells infiltrating adult valves.^43^ Heterogeneous macrophage lineages exist in the aortic and mitral valves of the heart during development and disease. These groups include resident macrophages of embryonic origin and macrophages of monocyte origin which are prevalent in diseases.^44^ During the development of the adult heart, heart valves occupy a variety of immune cell types. In addition to small populations of dendritic cells and T cells, at least 75% of these immune cells express macrophage markers that reside in and recruit populations.^45^ Therefore, exploring the unknown functions of macrophages in cardiac development, homeostasis, and disease is particularly crucial.

## Macrophage advances in various cardiovascular diseases

Macrophages have established themselves as the first line of defense against cardiovascular diseases.^46^ Targeting macrophages has emerged as one of the most effective treatments for various cardiovascular diseases, including pulmonary arterial hypertension (PAH), atherosclerosis, and MI. In the development of cardiovascular diseases, macrophages provide an irreplaceable contribution to the removal of senescent and dead cells, remodeling and plaque stabilization, and repair of myocardial injury. In Table 1, we summarize the molecular mechanisms of multiple macrophage phenotypic intervention targets for cardiovascular diseases in recent years.

**Table 1:** Novel targets of intervention modulate macrophage subtypes in various cardiovascular diseases.

Disease	Intervention target	Mφs subtype	Molecular mechanism	Reference
*Pulmonary arterial hypertension*				
Positive	Regnase-1	Alveolar-Mφs	Regnase-1 inhibits the development of pulmonary arterial hypertension in mice by controlling platelet-derived growth factors in alveolar macrophages.	47
Negative	Caspase-8	M1-Mφs	Caspase-8 promotes inflammatory cell infiltration and pulmonary artery smooth muscle cell proliferation via macrophage-mediated NLRP3/IL-1β activation.	48
Negative	MMP-10	M1-Mφs	STAT1 promotes the expression of MMP-10 in M1-polarized macrophages and promotes the proliferation and migration of pulmonary artery smooth muscle cells.	49
Negative	CCR2/CCR5	M2-Mφs	CCR2 and CCR5 synergistically promote M2-macrophage/pulmonary artery smooth muscle cell amplification of their migratory and proliferative capacities.	50
Negative	NLRP3	*CCR2*^*+*^-Mφs	NLRP3-macrophage activation mediates the cardiac inflammatory response and deterioration of cardiac function.	51
*Atherosclerosis*				
Positive	ALDH2	Resident-Mφs	ALDH2 interacts directly with Rac2 to inhibit Rac2 up-regulation and improve atherosclerotic plaques.	52
Positive	PPARβ	Monocytes-Mφs	Bone marrow-derived macrophage ppar β knockdown in mice increases pro-inflammatory genes and promotes the development of atherosclerosis.	53
Negative	PKM2	Monocytes-Mφs	Macrophage-specific knockdown of PKM2 reduces inflammatory gene expression and atherosclerosis by regulating LRP-1.	54
Negative	Gasdermin D	Resident-Mφs	Macrophage-specific inhibition of NLRP3/Caspase-1/amino terminal Gasdermin D suppresses macrophage focal death and IL-1β and IL-18 production, promoting atherosclerotic lesions.	55
Negative	Gasdermin E	Resident-Mφs	Inhibition of Gasdermin E in macrophages suppresses inflammation and macrophage pyroptosis.	56
Negative	HO1/ceruloplasmin/ferroportin	M1/M2-Mφs	HO1/ceruloplasmin/ferroportin are directly involved in macrophage polarization and iron transport and are regulated by inflammatory stimuli.	57
*Myocardial infarction*				
Positive	Osteopontin	*CD206*^*+*^-Mφs	IL-10-STAT3-galectin-3 regulates osteopontin expression and reparative macrophage polarization to promote myocardial repair.	58
Positive	Legumain	*CCR2*^+^-Mφs	Legumain deficiency enhanced CCR2^+^ Mφs and promoted the expression of pro-inflammatory mediators IL-1β, leading to cardiac dysfunction.	59
Positive	TIMD4	*CCR2*^*−*^-Mφs	Cx3cr1-based depletion post-infarct worsens cardiac function.	29
Positive	IL-35/IL-38	*Ly6C*^low^-Mφs	IL-35 and IL-38 improve ventricular remodeling by promoting the survival and polarization of reparative macrophages.	60 61
Negative	Mmp14	Resident-Mφs	Mmp14 activates transforming growth factor-β1 in macrophages and exacerbates cardiac fibrosis and adverse remodeling.	62
Negative	Dectin-1	M1-Mφs	Dectin-1-dependent IL-23/IL-1β production promotes IL-17a expression and exacerbates myocardial injury.	63
Negative	Lgr4	M1-Mφs	Lgr4 exacerbates myocardial injury by enhancing synergistic effects on inflammatory macrophage activator protein-1 activation via CREB.	64

## Regulation of macrophages in pulmonary arterial hypertension

PAH is considered the most prominent risk factor for cardiovascular diseases, and studies have shown that both innate and adaptive immunity can be involved in the pathogenesis of hypertension by triggering vascular inflammation and tissue remodeling.^65^ Classically activated macrophages, an essential component of the innate immune system, exacerbate the development of hypertension by secreting inflammatory cytokines that induce oxidative stress injury and endothelial dysfunction. Nevertheless, selectively activated macrophages have an active preventive and protective function in the development of hypertension. During the developmental phase of PAH, macrophage-derived caspase-8 plays a pathogenic role in peripulmonary vascular inflammation by promoting inflammatory cell infiltration and proliferation of pulmonary artery smooth muscle cells through the NACHT, LRR, and PYD domains-containing protein 3 (NLRP3)/interleukin (IL)-1β signaling activation.^48^ Regnase-1, also known as ZC3H12A and monocyte chemotactic protein-1, acts as an endoribonuclease responsible for the degradation of mRNAs involved in the inflammatory response.^66^ Regnase-1 maintains pulmonary innate immune homeostasis by controlling IL-6 and platelet-derived growth factor in alveolar macrophages, thereby inhibiting the development of PAH in mice.^47^ In patients with PAH, M1-polarized macrophages activate matrix metalloproteinase (MMP)-10, which regulates the malignant phenotype of pulmonary artery smooth muscle cells.^49^ However, migration of pulmonary artery smooth muscle cells to M2 macrophages was greater in PH patients and was attenuated by blocking CCR2 and CCR5. CCR2 and CCR5 are required between macrophages and pulmonary artery smooth muscle cells to synergistically initiate and amplify their migration and proliferation.^50^ Meanwhile, macrophage NLRP3 activates *CCR2*^*+*^ monocyte-derived macrophages, increases NLRP3 expression, and contributes to pulmonary hypertension-induced ventricular failure.^51^ Therefore, human circulating MMP-10, CCR2, CCR5, and NLRP3 can be used as a potential target therapeutic agent for PAH.

## Phenotype and function of macrophages in atherosclerosis

Atherosclerosis is a multifocal, inside-burning, lipid-induced inflammatory disease of the middle and large arteries involving immune cells, such as macrophages.^67^^,^^68^ Macrophage phenotype dysregulation is a primary trigger for atherosclerosis. In atherosclerosis, macrophage heterogeneity mainly includes M1, M2, and other phenotypes. It was shown that M1 and M2 macrophages accounted for 40% and 20% of total atherosclerotic lesion macrophages in mice, respectively.^69^^,^^70^ Macrophage subpopulations can be differentially polarized depending on the cytokines, chemokines, and growth factors present in the atherosclerotic plaque. NLRP3 inflammatory vesicles play an important role in macrophage pyroptosis, a pro-inflammatory cell death that is involved in the pathogenesis of atherosclerosis. Macrophage-specific inhibition of NLRP3/Caspase-1/Gasdermin D (GSDMD) signaling axis activation significantly reduced plaque area and IL-1β and IL-18 production, ameliorating macrophage inflammation and focal death-associated atherosclerotic lesions.^55^ However, transcriptome analysis of human atherosclerotic single cells showed that Gasdermin E (GSDME) was expressed mainly in macrophages. Mechanistically, GSDME deletion in macrophages inhibits oxidized low-density lipoprotein-induced STAT3 (signal transducer and activator of transcription 3) increase and macrophage scorching.^56^ In conclusion, characterizing GSDME- and Gasdermin D-mediated scorching in atherosclerosis progression may be a potential therapeutic approach for atherosclerosis. The glycolytic enzyme pyruvate kinase muscle 2 (PKM2) is up-regulated in monocytes/macrophages from patients with coronary atherosclerotic disease. Myeloid-specific deletion of PKM2 resulted in decreased inflammatory factors such as monocyte chemoattractant protein (MCP)-1, IL-1β, and IL-12, enhanced efferocytosis, and inhibited apoptosis and atherosclerosis.^54^ Similarly, aldehyde dehydrogenase 2 is a non-cytochrome P450 mitochondrial aldehyde oxidase that is associated with oxidative stress and mitochondrial dysfunction in many cardiovascular diseases.^71^ It interacts directly with Rac2 (Rac family small GTPase 2) to inhibit apoptotic cell-induced Rac2 up-regulation and subsequent efferocytosis, ameliorating atherosclerotic plaques.^52^ Nuclear receptor peroxisome proliferator-activated receptor (PPAR) was expressed in macrophages. Overexpression of PPARβ in mice increased the expression of MCP-1, MCP-3, IL-1β, and MMP-9, suggesting that PPARβ may also exert pro-inflammatory properties.^53^^,^^72^ However, PPARβ does not promote the M2 phenotype of human macrophages in human atherosclerotic lesions.^73^ These results suggest that PPARβ does not promote the M2 phenotype of human macrophages and does not correlate with CD206, C–C motif chemokine ligand 18, and IL-10 expression. During atherosclerosis progression, the mucoadhesive receptor CD146 activates macrophage activation by driving internalization of the scavenger receptor CD36.^74^ Genetic deletion of macrophage CD146 leads to migration of cholesterol-filled macrophages in the arterial wall and is a promising therapeutic target for atherosclerosis treatment. Ferroptosis is a novel form of cell death caused by iron-dependent lipid peroxidation. Recent studies have found that inflammation caused by iron death can promote the progression of atherosclerosis.^75^^,^^76^ Among these differentially expressed genes, heme oxygenase 1, ceruloplasmin, and ferroportin are directly involved in macrophage polarization and iron transport and are regulated by inflammatory stimuli. M1 macrophages exhibited iron transporter protein inhibition and free iron overload, promoting MMP secretion and leading to atherosclerosis plaque rupture. However, the up-regulation of iron transport proteins in M2 macrophages promotes iron release.^57^ The unique regulation of different phenotypic transitions in macrophages as iron homeostasis provides new perspectives and insights into their role in pathophysiological conditions.

## Macrophage repair in myocardial infarction

The heart contains abundant and heterogeneous macrophages, which are involved in the initiation of the inflammatory response and the reparative response through monocyte-derived macrophage recruitment and proliferation of cardiac macrophages after MI.^77^ Monocytes and macrophages are multipotent cells of the innate immune system and are essential in both the initial inflammatory response to cardiac injury and subsequent wound recovery. The monocyte-derived macrophage cells after MI, including pro-inflammatory M1 macrophages and repair M2 macrophages, strongly suggest significant heterogeneity and plasticity in macrophage development, phenotype, and function.^78^ Macrophages respond to changes in cytokine content and extracellular matrix composition and secrete fibrotic and angiogenic mediators that play a central role in the repair of the infarcted heart. Osteopontin and galectin-3 are associated with phagocytic clearance of dead cells and reparative fibrosis during wound healing. The IL-10-STAT3-galectin-3 axis is essential for the polarization of osteopontin-producing reparative *CD206*^*+*^ macrophages after MI, and these macrophages promote tissue repair by facilitating the clearance of fibrotic and apoptotic cells.^58^ However, post-MI originated from embryonic hematopoietic cardiac macrophages continuously clear and degrade apoptotic cardiomyocytes for inflammation regression and tissue repair. Legumain is a newly discovered cysteine protease belonging to the C13 peptidase family that is expressed mainly in macrophages.^59^^,^^79^ Cardiac macrophage legumain-specific deficiency significantly inhibited anti-inflammatory mediators IL-10 and transforming growth factor (TGF)-β, up-regulated pro-inflammatory mediators IL-1β, tumor necrosis factor-α, IL-6, and increased the infiltration of *MHC-II*^*high*^ *CCR2*^*+*^ macrophages and recruitment of *MHC-II*^*low*^ *CCR2*^*+*^ monocytes. Tissue-resident *CCR2*^*+*^ macrophages after cardiac injury promote monocyte recruitment through a myeloid differentiation primary response-dependent mechanism, leading to monocyte chemotactic protein release and monocyte mobilization.^59^ In contrast, tissue *CCR2*^*−*^ macrophages inhibit monocyte recruitment. Meanwhile, ischemic injury reduced the abundance of T-cell immunoglobulin and mucin domain containing 4 (*TIMD4*)^*+*^ and *TIMD4*^*−*^ resident macrophages, whereas *CCR2*^*+*^ monocyte-derived macrophages adopted multiple cell fates within the infarcted tissue, revealing a non-redundant cardioprotective role for resident cardiac macrophages.^29^ Dectin-1 expression is increased in bone marrow-derived macrophages in the heart in the early stages after cardiac injury. Dectin-1-dependent IL-23/IL-1β production promotes IL-17a expression, leading to neutrophil recruitment and myocardial injury.^63^ Macrophage production of Mmp14, a factor involved in cardiac repair and remodeling after MI, mediates the repair of other cell-type crosstalk mechanisms that have been progressively elucidated. Researchers discovered that Mmp14 activates TGF-β1 in macrophages, leading to SMAD family member 2-mediated paracrine signaling in endothelial cells and endothelial–mesenchymal transition, which exacerbates cardiac fibrosis and adverse remodeling.^62^ In addition, IL-35 and IL-38^60^^,^^61^ reduce post-infarction cardiac rupture and improve post-infarction ventricular remodeling by promoting the survival of reparative *CX3CR1*^*+*^ *Ly6C*^*low*^ macrophages and regulating macrophage polarization. Interestingly, deletion of the macrophage-associated immunomodulatory factor Lgr4 (leucine-rich repeat-containing G protein-coupled receptor 4) reduced the number of infiltrating leukocytes and inflammatory macrophages at the site of MI but enriched the subpopulation of reparative macrophages.^64^ Notably, Lgr4 exacerbates myocardial injury by enhancing the synergistic effect of cyclic adenosine monophosphate response element binding protein on activator protein-1 activation of inflammatory macrophages. In conclusion, several findings highlight the critical process by which numerous immune-related regulatory factors control the pro-inflammatory phenotype of infarcted macrophages and post-infarct repair.

## Macrophages in cardiomyocyte proliferation and regeneration

**Figure 2:**
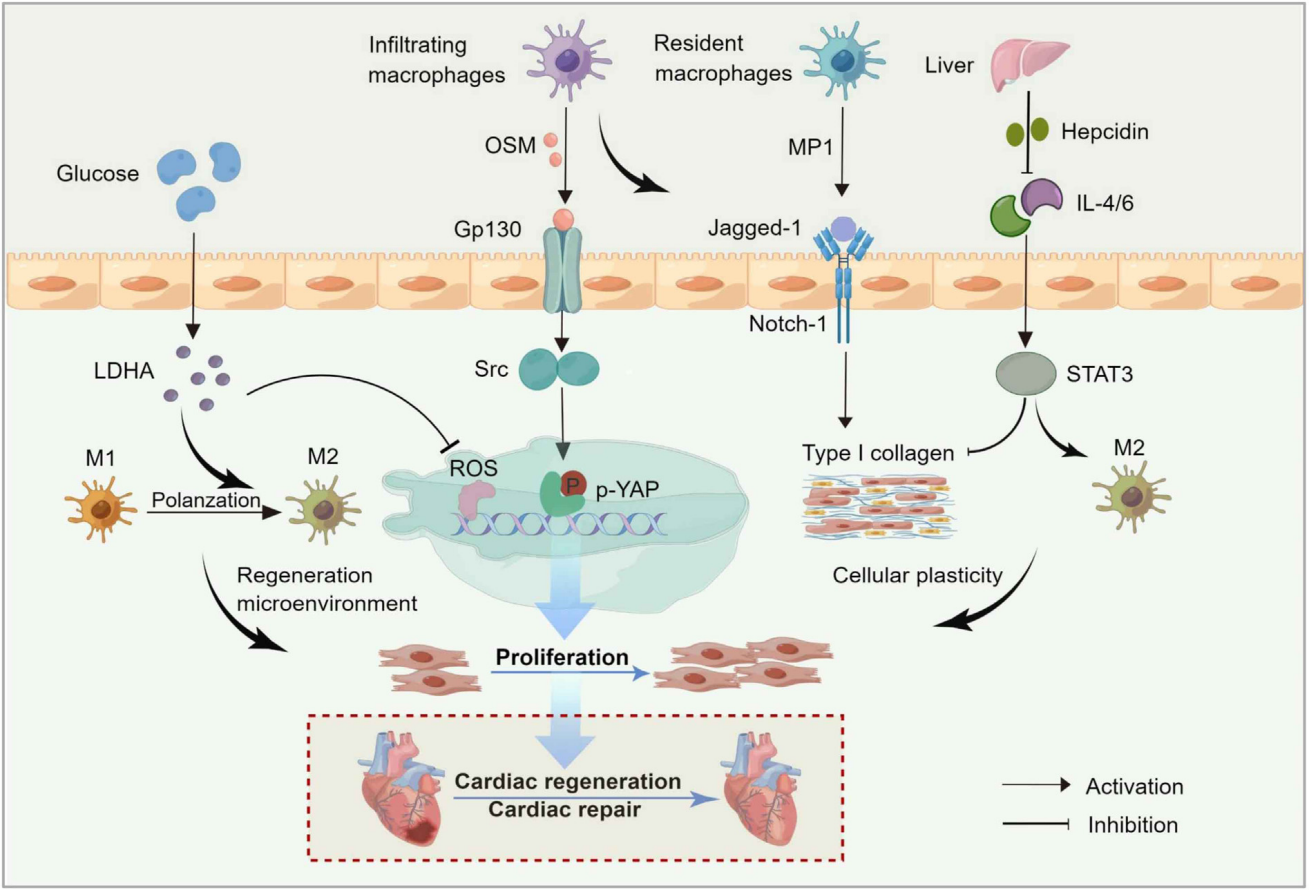
Schematic representation of cardiac macrophages initiating cardiomyocyte proliferation and repair. During the inflammatory response to myocardial injury, oncostatin M (OSM) secreted by cardiac infiltrating macrophages binds to the type II receptor Gp130 and stimulates Src-enhanced YAP phosphorylation to promote cardiomyocyte proliferation and myocardial repair. Meanwhile, the cardiac resident macrophage subpopulation MP1 (*CX*_*3*_*CR1*^+^*CCR2*^*−*^*Ly6C*^*−*^*MHCII*^*−*^) directly triggered cardiomyocyte proliferation via the Jagged-1-Notch1 axis, significantly ameliorating cardiac injury after myocardial infarction. The glycolytic metabolite lactate dehydrogenase A (LDHA) creates a beneficial cardiac regenerative microenvironment through the polarization of M2 macrophages. The iron-regulating hormone hepcidin and interleukin (IL)-4/6 enhance cellular plasticity and adaptation of the extracellular matrix microenvironment, facilitating cardiac regeneration and repair.

Various cardiovascular diseases are the main cause of morbidity and mortality in many countries around the world.^80^ Heart diseases, characterized by myocardial defects, can cause severe cardiac dysfunction and eventually develop into heart failure. As a rapidly growing global public health problem, heart failure poses a heavy burden on the quality of life and medical expenses of patients. Macrophages, as key effectors of cellular immune inflammation, play a particularly important role in cardiovascular diseases.^81^ Resident macrophages are thought to have important functions in the organization and maintenance of homeostasis in many organs, including the brain, liver, adipose tissue, lymphoid tissue, and the intestine.^25^ However, the mechanisms by which cardiac-resident macrophages initiate myocardial proliferation and repair are still being explored. Cardiac M1 macrophages clear debris produced by damaged cardiomyocytes and promote the inflammatory response after cardiac injury.^82^ M2 macrophages are alternately activated and exhibit anti-inflammatory and myocardial repair phenotypes under pathological conditions. After cardiac apical resection on neonatal mice, myocardial tissue will regenerate completely within 21 days, but this situation is limited to P7 mice. Multiple studies have shown that adult mouse cardiac monocytes/macrophages trigger the formation of myocardial fibrosis and scarring.^83^^,^^84^ In contrast, neonatal mouse macrophages differ from adult mice in polarization patterns and transcriptional profiles, and the differences in transcriptional profiles reflect differences in gene function. This suggests a unique pattern of cardiac regeneration mediated by neonatal mice. Understanding the mechanisms of macrophage-dependent cardiac regeneration is a potential strategy to enhance cardiac repair (Fig. 2).

## Mammalian macrophages and cardiomyocyte proliferation

To understand the difference between the immune response during and after regeneration, the researchers profiled the cellular response to MI in P1 and P14 mice over time using fluorescence-activated cell sorting.^8^ Compared with P14, the abundance of F4/80^+^ macrophages in the heart of P7 mice after MI was relatively increased. However, the depletion of cardiac macrophages in Cl_2_MDP-L-treated mice severely affects cardiac tissue regeneration in P7 mice and contributes to significant scarring. Notably, neonatal mice develop distinct pathological features early in myocardial injury, including blood clot formation and acute inflammatory responses. The development of acute inflammation accompanied by regeneration of myocardial tissue predicts that immune regulation plays a key role in initiating myocardial regeneration in neonatal mice.

Oncostatin M (OSM), a pleiotropic cytokine and a member of the glycoprotein 130 (Gp130) family cytokines, plays a significant role in inflammation, autoimmunity, and cancers.^85^^,^^86^ Studies have shown that OSM secreted by macrophages stimulates inflammatory gene expression in cancer-associated fibroblasts, which in turn induces a pro-tumor environment and participates in tumor cell survival and migration signaling pathways.^87^ Similarly, during the onset of injury in the neonatal mouse heart, OSM is recruited to sites of inflammation primarily through paracrine secretion by macrophages. Through RNA sequencing and fluorescence-activated cell sorting, OSM is mainly derived from cardiac macrophages^11^ and is highly expressed after neonatal mouse heart one-day post-resection. OSM derived from cardiac macrophages binds to the type II receptor Gp130^88^, which promotes cardiomyocyte proliferation and myocardial repair by stimulating Src to enhance Yap (Y357) physiology independently of the Hippo pathway during cardiac regeneration. In summary, myocardial injury induces the synthesis of OSM in cardiac macrophages, which plays a key and sustained role in mediating the regulation of multiple cardiac regeneration signaling pathways by Gp130. Myeloid-derived growth factor is a paracrine protein produced by bone myeloid-derived monocytes and macrophages and is mainly expressed in *Ly6C*^*high*^ monocytes, *Ly6C*^*low*^ monocytes, or mononuclear macrophages, T cells, B cells, endothelial cells, and cardiomyocytes.^89^, ^90^, ^91^ Employing RNA sequencing and functional verification, myeloid-derived growth factor promotes cardiomyocyte proliferation and cardiac regeneration in adult mice after MI by activating the c-Myc/Forkhead box M1 pathway, providing a potential target for reversing cardiac remodeling and heart failure. Macrophages with different activation states produce a variety of growth factors, such as insulin-like growth factor 1, vascular endothelial growth factor (VEGF)-α, TGF-β, and Wnt proteins, which regulate epithelial and endothelial cell proliferation, myofibroblast activation, progenitor cell differentiation, and angiogenesis.^92^ Metabolic transitions during cardiac development regulate postnatal cardiomyocyte cycle exit and cardiac regenerative capacity in the mammalian heart. It was demonstrated that lactate dehydrogenase A creates a beneficial cardiac regenerative microenvironment by inducing metabolic reprogramming through polarization of M2 macrophages.^93^ It has emerged as an effective intervention target for cardiac repair after MI.

The highly heterogeneous population of cardiac-resident macrophages plays an important role in maintaining cardiac homeostasis, development, and remodeling. Recently, the dynamics of cardiac resident macrophages during individual myocardial development were monitored by single-cell sequencing technology to analyze their phenotypic and functional properties in promoting cardiac regeneration.^10^ During mouse heart development, four cardiac-resident macrophage subpopulations are sequentially present: MP1, *CX3CR1*^*low*^ *CCR2*^*low*^ *Ly6C*^*−*^ *MHCII*^*−*^ (MP2), *CX3CR*^*−*^ *CCR2*^*+*^ *Ly6C*^*−*^ *MHCII*^*−*^ (MP3) and *CX3CR1*^*+*^ *CCR2*^*−*^ *Ly6C*^*−*^ *MHCII*^*+*^ (MP4). Embryonic and neonatal-derived MP1 directly triggers cardiomyocyte proliferation via the Jagged-1-Notch1 axis, significantly ameliorating cardiac injury after MI. The MP2/3 isoform survives throughout adulthood. MP4 is the major population in the adult mouse heart, leading to inflammation. During individual development, MP1 can be triggered by changes in cellular redox status to MP4. These findings describe the evolutionary kinetics of cardiac-resident macrophages under physiological conditions and uncover direct evidence that cardiac-resident macrophages of embryonic and neonatal origin regulate cardiomyocyte proliferation.

## Macrophages and cardiomyocyte proliferation in zebrafish

Unlike mammals, zebrafish have a strong ability to restore tissue integrity, structure, and function.^94^, ^95^, ^96^ After the occurrence of cardiac injury, adult mammals can only promote the repair of maladaptive disorders, while zebrafish compensate for the loss of cardiac muscle cells through the dedifferentiation and proliferation of cardiac muscle cells to achieve complete cardiac regeneration.^97^^,^^98^ Cardiac regeneration is a complex process that is accompanied by transient fibrosis, blood supply remodeling, and inflammatory reactions.

Macrophages directly promote collagen scar formation during zebrafish heart regeneration and mouse heart repair.^99^ In particular, macrophages have been shown to eliminate the regeneration of multiple organs and organisms and become an important cellular regulator of cardiac regeneration. Recent studies confirm the macrophage-dependent regulation of adrenergic signaling in zebrafish cardiomyocyte proliferation and repair.^100^ Adrenergic receptor alpha-1 induces macrophage phenotypic switching and extracellular matrix remodeling after myocardial injury in zebrafish. Zebrafish macrophages promote the formation of cardiac regenerative microenvironmental niches through midkine-mediated paracrine crosstalk, leading to lymphatic and vascular growth and cardiomyocyte proliferation at the site of injury. These findings identify macrophage phenotypes and functions that mediate adrenergic signaling mechanisms and regulate fibrosis and myocardial regenerative responses. Additionally, macrophages invade the epicardial myocardial niche, inducing an increase in the number of epicardial cells, thereby increasing the expression of VEGF in the epicardial membrane, leading to an upregulation of the endocardial notch signaling pathway, thereby exerting its proliferative effect.^101^ Therefore, the discovery of macrophage-dependent cardiac regeneration mechanisms supports immune regulation as a promising strategy for enhancing mammalian heart repair.

## Interleukin and cardiac regenerative therapies

Research on direct induction of endogenous myocardial proliferation and cardiac regeneration has progressed and is a potentially effective strategy for the treatment of cardiovascular diseases. As effective regenerative compensators, targeting immune cells and inflammatory responses are considered to be powerful regulators of cardiac regeneration. Myocardial injury inevitably triggers a severe inflammatory response, leading to immune cell recruitment, myocardial damage, and scar formation. The nature of the early postnatal immune response in rodents appears to influence cardiac regeneration and the underlying molecules are still being explored. Using cytokine arrays, IL-4 and IL-6 expressed in M2 macrophage-conditioned medium induced endothelial cell proliferation, whereas IL-4 promoted neonatal cardiomyocyte proliferation and blocked myofibroblast-induced type I collagen secretion.^102^ Morimoto H et al found that MCP-1 induces macrophage infiltration, angiogenesis, myocardial IL-6 secretion, and myocardial fibroblast accumulation, thereby improving the prevention of myocardial remodeling after MI.^103^^,^^104^ Thus, the immune inflammatory factor MCP-1 significantly shortened the inflammatory phase and weakened left ventricular remodeling. Interestingly, there is a strong coupling between the developmental characteristics of cardiomyocytes and the maturation of the immune system, suggesting that immune cells may affect the proliferative ability of mammalian cardiomyocytes. However, these studies strengthen the link between immunology and cardiac regeneration and provide far-reaching implications for cardiac regenerative therapies.

## Iron metabolism and macrophage-dependent cardiac regeneration

Hepcidin, an iron-regulating hormone, is secreted by hepatocytes and acts by controlling the activity of cellular iron transport proteins that absorb iron from the intestine and transport it to the plasma.^105^, ^106^, ^107^ As a regulator of systemic iron homeostasis, its functions include plasma iron concentration, body iron stores, infection and inflammation, and erythropoiesis. In recent years, studies have demonstrated that defects in systemic and local iron metabolism are associated with cardiac disease and play a crucial role in the regulation of cardiac homeostasis after MI. The study has identified a significant increase in hepcidin expression after acute MI.^108^ In the context of myocarditis and MI, hepcidin is strongly induced in cardiomyocytes with elevated levels of inflammatory cytokines that may play an important role in iron homeostasis and free radical generation.^109^, ^110^, ^111^ Interestingly, cardiomyocyte-specific deletion of hepcidin did not improve cardiac repair and function. However, bone marrow cell-derived hepcidin-specific deletion mice had substantially reduced infarct size and tissue fibrosis and increased cardiomyocyte renewal.^108^ Moreover, macrophages lacking hepcidin promote cardiomyocyte proliferation in a model of apical resection-induced cardiac regeneration in neonatal mice. In cardiomyocytes, hepcidin deficiency enhanced the number of *CD45*^*+*^*/CD11b*^*+*^*/F4/80*^*+*^*/CD64*^*+*^*/MHCII*^*low*^*/CCR2*^*+*^ inflammatory macrophages and promoted STAT3, which in turn released IL-4 and IL-13 and did not improve cardiac function in adult and neonatal injured hearts. IL-4/IL-13 released by cardiac macrophages accelerates cardiac regeneration and improves myocardial remodeling. Therefore, this tissue injury-induced interleukin release can promote cell plasticity and the adaptation of the extracellular matrix microenvironment conducive to tissue repair.

## Macrophages induce neovascularization and myocardial regeneration

Angiogenesis and repair are crucial in MI due to coronary artery disease.^112^, ^113^, ^114^ Therefore, activation of neovascularization is an innovative strategy and a major challenge to restore nutritional supply to the infarcted myocardium. Studies have indicated that non-classical monocytes and reparative macrophages become the turning point between inflammation and its ablation, thus controlling repair and angiogenesis.^115^^,^^116^ M1-type macrophages play a critical role in angiogenesis and cardiac repair after MI. M1-like macrophage-derived exosomes increase miR-155 release and inhibit Sirtuin 1/AMP-activated protein kinase α2-endothelial nitric oxide synthase and RAC1-p21-activated kinase 2 signaling pathways, thereby inhibiting angiogenesis and cardiac function. Membrane-linked protein A1 (AnxA1) is rapidly released in response to cellular stress and blocks chemokine-mediated integrin activation, thereby shutting down inflammatory recruitment of myeloid cells.^117^^,^^118^ Cardiac macrophages from AnxA1-deficient mice reduced the release of VEGF-A and decreased angiogenic capacity. However, AnxA1 treatment enhanced VEGF-A release from cardiac macrophages. Bone marrow-derived *Cx*_*3*_*cr1cre*^*ERT2*^ *Vegf*^*flox/flox*^ or depleted macrophages in mice reversed the positive effects of AnxA1 treatment on cardiac performance. In conclusion, AnxA1 acts directly on the polarization of cardiac macrophages toward a pro-angiogenic, repair phenotype.

## Macrophages and human-induced pluripotent stem cells

Human pluripotent stem cells such as embryonic stem cells and iPSCs offer unprecedented opportunities for cellular therapy of recalcitrant cardiovascular diseases.^119^ Meanwhile, iPSCs have made a valuable contribution to the field of regenerative medicine, paving the way for identifying the true potential of human embryonic stem cells.^120^^,^^121^ Studies have shown that iPSC-derived cardiomyocytes can be produced and expanded in large numbers *in vitro* by being delivered to the injured heart.^122^ The macrophage phenotype shifts from M1 to M2 after myocardial injury.^8^ However, iPSCs implanted for myocardial repair inevitably come into contact with the inflammatory environment at the site of MI. That said, there are a number of practical issues that limit its use, including its inherent tumorigenicity, immunogenicity, and heterogeneity. Different macrophage subtypes regulate the proliferation, cardiac differentiation, and maturation processes of iPSCs. Cardiomyocyte formation was significantly inhibited by co-culturing M0, M1, and M2 macrophages with iPSCs. Notably, compared with M0 and M2 macrophages, M1 macrophage co-culture significantly reduced the proliferation, cardiac differentiation, and maturation of iPSCs.^12^ In conclusion, macrophages play a key role in the proliferation and myogenesis of iPSCs and provide the necessary guidance for the implantation of iPSCs into infarcted hearts for cardiac regenerative therapy.

## Conclusion and future perspective

In cardiac disease, homeostatic cardiac macrophages, monocyte-derived macrophages, and tissue-localized macrophages express different genes in a pattern different from the M1/M2 macrophage polarization paradigm.^123^ The transformation and homeostasis of the human cardiac macrophage pool regulates myocardial regeneration and repair, profoundly affecting the progress of cardiovascular disease treatment. This review spotlights recent research advances in macrophage fate specification and heterogeneity and cardiac macrophage research in cardiomyocyte proliferation. Monocytes and macrophages are essential components of the immune system and, as the critical effectors and regulators of the innate immune response, they have critical and unique physiological functions in cardiac development and homeostasis. Heterogeneity and plasticity characterize the monocyte-macrophage lineage. In tissue repair and regenerative tissues, monocyte-derived macrophages and cardiac tissue macrophages and their paracrine factors influence M1 and M2 phenotypic transitions.^124^ Monocytes typically originate from progenitor cells in the bone marrow and are transported through the bloodstream to the peripheral blood and surrounding tissues. During homeostasis and inflammation, circulating monocytes differentiate into macrophages or dendritic cell populations in response to local growth factors, pro-inflammatory cytokines, and microbial products. The recruitment of monocytes effectively controls and clears viral, bacterial, fungal, and protozoal infections, however, recruited monocytes also contribute to the development of inflammatory and degenerative diseases.^124^, ^125^, ^126^, ^127^ After birth, bone marrow-derived monocytes can replenish tissue macrophages following injury, infection, and inflammation.

The heterogeneity of macrophage lineages has been widely recognized, in part due to the specialization of macrophages in specific tissue microenvironments. However, the origin of tissue macrophages, which are critical for homeostasis and immunity *in vivo*, remained controversial until recently. Fate profiles elucidate the cellular origin of macrophages within developing cardiac tissue. Genetic lineage tracing systems revealed little contribution of the endocardium to cardiac macrophages and circulating blood cells. During embryonic organogenesis, macrophages from the YS and fetal liver precursors are colonized throughout the tissues and continue to function physiologically as resident, self-sustaining populations during adulthood. In essence, elucidating the developmental origin of cardiac macrophages and their function in cardiac development will advance our understanding of cardiac development, pathogenesis, and regeneration.

Cessation of cardiomyocyte renewal after cardiac injury is a major cause of high mortality from cardiovascular diseases. It takes an effective position in maintaining cardiac homeostasis by initiating cardiac macrophage-specific gene expression to protect the organism from pathogens, eliminating apoptotic cells, and promoting myocardial regeneration and repair regulation in a state of myocardial injury. Massive recruitment of macrophages at the site of MI and secretion of endogenous regulatory factors directly initiate cardiomyocyte proliferation and myocardial regeneration. During cardiac development, disturbances in the function of the lymphatic vascular system can lead to myocardial edema and a sustained inflammatory response.^128^ Therefore, the interaction between cardiac lymphocytes and macrophages during myocardial regeneration and repair after MI deserves further investigation. The relative contribution of macrophages in the myocardial repair response before and after the closure of the regenerative regeneration window was progressively resolved using RNA sequencing with single-cell sequencing data. Non-regenerating hearts generate more inflammation-related stromal cell responses, recruiting specialized macrophages to express higher levels of risk-associated molecular pattern receptors. Metastasis of individual macrophages in the heart after birth may lead to deleterious stress signals that inhibit cardiac regeneration. The mammalian postnatal heart is capable of regenerating immediately after infarction, but the molecular mechanisms of signaling that regulate this behavior are not fully understood. Cardiomyocyte proliferation as a promising strategy for repairing cardiac function requires an urgent understanding of the molecular mechanisms targeting immune cell-mediated cardiac regeneration, particularly macrophages.

## Author contributions

T.W. wrote the original manuscript; T.W., X.W., W.R., and Y.Z. designed the figures; Z.S., N.W., and H.D. revised the manuscript; all authors approved the final manuscript.

## Funding

This work was supported by the 10.13039/501100012166National Key Research and Development Program of China (No. 2021YFA1301100, 2021YFA1301101), 10.13039/501100007129Shandong Provincial Natural Science Foundation of China (No. SYS202202), and the Research Project of Jinan Microecological Biomedicine Shandong Laboratory (China) (No. JNL-2023009Q, JNL-2022012B).

## Conflict of interests

The authors declared no conflict of interests.
